# Implementing a psycho-educational model to increase university lecturers’ effectiveness to constructively manage experienced aggression

**DOI:** 10.4102/hsag.v25i0.1364

**Published:** 2020-12-02

**Authors:** Rika R. Toerien, Chris P.H. Myburgh, Marie Poggenpoel

**Affiliations:** 1 Department of Educational Psychology, Faculty of Education, University of Johannesburg, Johannesburg, South Africa; 2 Department of Nursing Science, Faculty of Health Sciences, University of Johannesburg, Johannesburg, South Africa

**Keywords:** experienced aggression, implementation, lecturers, psycho-educational model, increased effectiveness, university workshop

## Abstract

**Background:**

The authors developed a psycho-educational model as a conceptual framework of reference for university lecturers to facilitate the constructive management of experienced aggression. The model must be implemented in a workshop and in practice to confirm the if the model is effective.

**Aim:**

This article describes the implementation of a psycho-educational model in a workshop and in practice, as well as the evaluation of the effectiveness of the psychoeducational model.

**Setting:**

This study was conducted in a specific college at a university in Johannesburg in South Africa.

**Method:**

This study followed a qualitative, exploratory, descriptive, contextual and theory-generating research design. The psycho-educational model was implemented in three phases during a workshop and then for three months in practice by university lecturers. A purposive sample of university lecturers was applied. The effectiveness of the psycho-educational model was evaluated during and directly after the workshop, 1 week after the workshop and 3 months journal entries. Final evaluation was in a focus group after 3 months of implementation of the model in the workplace.

**Results:**

The participating university lecturers found the implementation of the psycho-educational model, as a conceptual framework of reference to constructively manage experiences of aggression, effective, helpful and important. The model increased their understanding of aggression in their places of work and increased their effectiveness to constructively manage experiences of aggression in their workplace.

**Conclusion:**

The implementation and evaluation of the psycho-educational model underscored the need for affective and effective facilitative support for university lecturers to be able to constructively manage experienced aggression.

## Introduction

Aggression in the workplace is a globally destructive phenomenon that breaks down interaction, communication and relationships if it is not addressed and constructively managed. Liberman (2016:n.p.) asserted that aggressive behaviour at work has progressively become damaging for the overall climate in the workplace. Aggression in the workplace assumes a variety of forms and is used for various reasons by individuals or groups (Breet, Myburgh & Poggenpoel 2010:514–515; Kelloway et al. 2010:18–19). It affects human beings of all ages, cultures and gender and is expressed in a variety of behaviours (Breet et al. 2010:511). DeWall, Anderson and Bushman (2011:245) stated that aggression often results in reciprocal aggression and causes more problems than solutions. Conflict that is not managed effectively may have immediate and long-lasting effects on a person (Einarsen, Mikkelsen & Matthiesen n.d:1). Employees who receive support from their organisations frequently experience feeling more in control and express a positive frame of mind (Everton, Jolton & Mastrangelo 2009:53). As a result, employees who are in control will experience better job satisfaction, professional effectiveness and overall well-being (Loh, Restubog & Zagenczyk 2010:236).

Unfortunately, universities did not escape this social tendency and phenomenon, and support is needed to increase university lecturers’ effectiveness in dealing with and controlling experiences of aggression at work (Toerien 2014:64–101). A considerable amount of literature has been published on models that address aggression in, *inter alia*, schools, nursing, amongst students and in the workplace, in general (Bimenyimana 2015; Botha 2006; Evangelides 2007; Jacobs 2013; Mbadi 2009). However, relatively few studies have been published on university lecturers’ experiences of aggression, or on models and intervention programmes for university lecturers to develop their effectiveness to constructively manage their experiences of aggression.

**TABLE 1:** Three phases of the facilitation process of model implementation.

Phases	Purpose with reference to the model
Relationship phase	Build a relationship and trust Understand the problem Establish shared objectives
Working phase	Facilitate constructive management of experienced aggression: discover, explore and describe knowledge and skills on intra- and inter-personal attributes and competencies, communication and conflict management skills that are helpful to promote university lecturers’ development to manage experienced aggression constructively and increase their effectiveness in dealing with and controlling experiences of aggression
Termination phase	Retrospective, introspective and summative reflection on the process and learning Evaluate the implementation of the model and increased effectiveness and growth to implement the model in practice to ‘manage’ experiences of aggression ‘constructively’

*Source:* Adapted from Dickoff, J., James, P. & Wiedenbach, E., 1968, ‘Theory in a practice discipline - part 1. Practical-oriented theory’, in L.H. Nicoll (ed.), *Perspective on nursing theory*, pp. 415–435, Little Brown & Company, Boston, MA

The authors in this article attempted to bridge this identified gap in the existing body of knowledge by developing a ‘psycho-educational model for university lecturers to facilitate constructive management of experienced aggression’ (Toerien 2019:69–137), which was applied in a workshop for the purposes of this article. Dickoff, James and Wiedenbach (1968:131–135) underscored the importance of theory description and clarification within operationalisation, implementation and evaluation. Therefore, this article describes the implementation and evaluation of a developed psycho-educational model (Toerien 2019) for university lecturers to facilitate the constructive management of experienced aggression.

## Scientific and methodological contextualisation

The psycho-educational model was implemented in a workshop and then at the lecturers’ places of work. The primary goals of the workshop were to firstly implement a developed psycho-educational model for university lecturers to facilitate the constructive management of experienced aggression and secondly to evaluate the effectiveness of the psycho-educational model as a facilitative and conceptual framework of reference for university lecturers to constructively manage the experiences of aggression. The implementation of the psycho-educational model in the workshop required the facilitator to assist the university lecturers to increase their effectiveness to manage experiences of aggression constructively. This increased effectiveness may be helpful to them and may promote their development. Also, university lecturers’ promoted development and increased effectiveness may increase their ability to deal with and control their experiences of aggression in their places of work. After the workshop, the lecturers implemented the psycho-educational model in their places of work followed by a focus group to finally evaluate the effectiveness of the implementation of the psycho-educational model.

## Method

### Psycho-educational model implementation

The psycho-educational model was implemented in three phases during a workshop: phase 1 – the relationship phase, phase 2 – the working phase and phase 3 – the termination phase. The implementation process established a single case study at a university in South Africa (Creswell 2013:97; Gustafsson 2017:11; Heale & Twycross 2018:7). Rule and John (2015:3) clarified a case study to be distinctive and specific. In this research, the single case study was an instrumental case study selected to evaluate the psycho-educational model’s effectiveness through implementation in a workshop. The facilitation process of the model postulated an active and engaged process with the focus on increasing university lecturers’ effectiveness in managing aggression more constructively. According to Belmont (2017:n.p.), the participants’ effectiveness may increase in a workshop setting because they are presented with opportunities to develop, communicate, collaborate, learn from others and acquire skills and personal self-discovery and growth in a social context with feedback and support.

After the workshop, the lecturers implemented the psycho-educational model and skills developed during the workshop at their workplaces.

### Population and sample

The purposive sample for the workshop in this study included university lecturers who were academic faculty members within a specific university in South Africa. The inclusion criterion was that participants must have been working within the faculty for at least 3 years, but not more than 5 years. Twenty-eight potential participants were identified for the workshop from the data presented to the researchers by the specific academic division. From a total of 28 university lecturers who met the participation criteria, 7 accepted the workshop invitation. Five of the seven invited participants attended the workshop. Of the seven participants, three were women and two were men. Two participants were between the ages of 40 and 50 years and three were between the ages of 30 and 40 years. The participants also represented the cultural diversity of the university and country.

## Evaluation during and after the workshop

The university lecturers were assisted to increase their effectiveness in managing experiences of aggression constructively through knowledge and skills discovery, reflection, as well as meaning-making and problem-solving. The workshop process was audio-recorded, followed by a verbatim transcription of the audio recording. Participants also completed written reflective feedback directly after the workshop on one open-ended question, ‘how was the model implementation in the workshop for you?’ In conclusion of the psycho-educational model’s implementation in a workshop, participants had to complete a reflective journal entry 1 week after the workshop. Participants answered one open-ended question: ‘reflecting back on the workshop, how was the model implementation in the workshop for you?’ Additionally, the presenting researcher’s (R.R.T.) observations and field notes contributed to the evaluation.

## The facilitation process and workshop programme to implement the model

The model was implemented at the university where the participants worked and experienced aggression. The workshop consisted of a single, half-day programme. The psycho-educational model’s implementation in this workshop seeks to address the primary question, ‘what can be done to support university lecturers to constructively manage experienced aggression?’ The psycho-educational model’s implementation is based on a facilitative process that the facilitator makes easier for the university lecturers to participate and develop skills. The facilitative workshop programme followed three phases sequentially: the relationship phase, the working phase and the termination phase. These three phases directed the workshop design and facilitation process, with a variety of instructional activities and opportunities for reflection, dynamic and collaborative interaction and participation for discovery and self-directed learning.

To apply theoretical prescriptions to practice is complex, but facilitation is a promising technique to successfully apply theory into practice (Cranley et al. 2017:1). The facilitation process of the model postulated an active and engaged process with the focus on increasing university lecturers’ effectiveness in managing aggression more constructively. The facilitator engaged the university lecturers who experienced aggression in a facilitation process to make it easier for them to participate and communicate in the workshop. The facilitator assisted the university lecturers to increase their effectiveness in managing their experiences of aggression. University lecturers’ increased effectiveness could be helpful to them and promote their development in dealing with and controlling their experienced aggression. The process allowed progress divergence to accommodate the potential need for additional assistance based on university lecturers’ uniqueness and individuality. The outcome of the psycho-educational model was the guiding principle in the implementation of the workshop, namely, university lecturers’ increased effectiveness in the constructive management of experienced aggression.

### Phase 1: Relationship phase

The relationship phase in the process of the psycho-educational model was fundamental for the successful implementation of the model and critical for participants to progress towards the working phase and the termination phase. This phase established the starting point for a trusting relationship, active participation and dynamic and collaborative interaction that the rest of the process depended on. The main objectives of this phase were (1) to build a relationship and trust to assist the university lecturers and make it easier for them to participate in the ‘facilitation’ process, (2) for participants to understand the phenomenon of aggression experienced in higher education and the problem of destructive management of experienced aggression and (3) for the group to establish (a) individual objectives and (b) shared objectives.

### Phase 2: Working phase

In this article, to ‘facilitate the constructive management’ of experienced aggression is defined as a process that the facilitator makes easier for university lecturers. The facilitator assisted the university lecturers to increase their effectiveness in constructively managing their experiences of aggression. The university lecturers’ increased effectiveness could promote their development, which could enable them to deal with and control the experienced aggression. During the working phase, the facilitator’s direct involvement decreased to allow participants to enhance their effectiveness to ‘constructively’ ‘manage’ experienced aggression. This was achieved through a dynamic and collaborative interaction, as well as self-directed learning, discovery, knowledge and skills development. Participants had to engage in understanding and meaning-making of the concepts ‘constructive management’ and find solutions for their experiences of aggression that would be helpful to them and could promote their development in dealing with and controlling these experiences. University lecturers achieved personal and professional growth and development through the implementation of individual, subgroup and group activities to increase their knowledge and skills on an intra- and inter-personal level. They also gained improved communication and conflict management skills.

### Phase three: Termination phase

In the termination phase, firstly, the main objectives were a retrospective, introspective and summative reflection on the implementation process of the model in the workshop and individual learning and development on a personal and professional level. Secondly, participants reflected as a group on whether their shared objectives were met through the implementation of the model in the workshop, thus increasing their effectiveness in implementing the model in practice in their workplace. Thirdly, participants evaluated the implementation of the model in the workshop and their increased effectiveness and growth in implementing the model in practice at their places of work. Finally, participants submitted individual written responses that contributed to the results of the research. An agreement was reached by the university lecturers to implement the model in practice in their own work environment.

The workshop objectives and design denoted the implementation of the model. The facilitator used various tasks and activities – each with a specific purpose – during the three phases of the workshop to achieve the objectives and the aim of the model, namely, the constructive management of experienced aggression by university lecturers. The tasks, activities and purpose of the activities are presented in Table 2.

**TABLE 2:** Tasks, activities and purpose of the workshop phases and programme in relation to the model.

Task	Activities	Purpose
**Phase 1: Relationship phase**		
Building trust	University lecturers who were willing to share told the groups something nobody knows about them	To build participants’ trust and confidence to increase their ability for discovery and learning in a group
Defining aggression	Participants had to visualise how they view the term ‘aggression’ and then discuss it as a group	Understanding the phenomenon of aggression experienced in higher education
Defining the problem	Three pictures of destructive behaviour. Problem identification	Understanding destructive management of experienced aggression
Shared objectives	Shared group objectives from individual objectives	Identify individual goals as well as collaborative group decision-making and goals
**Phase 2: Working phase**		
Understanding of central concepts ‘constructive’ and ‘management’	Central concept puzzle building Three open-ended questions for meaning-making on essential attributes that are helpful and that promote development to increase effectiveness Two open-ended questions for meaning-making on essential attributes to deal with and control experienced aggression	Critical thinking and meaning-making of the concepts Insight and cognisance development
Personal responses to aggression experiences	Show images of various types of aggression and self-reflect on own behaviour	Self-reflection on personal aggression management skills and behaviour
Knowledge and skills development to increase effectiveness	List personal internal strengths and weaknesses Tick and add interpersonal competencies Reflecting and recalling participants’ information on something nobody knows Describing pictures with various meanings Brief collaborative discussion: how will you implement new knowledge and skills to achieve constructive management?	Increase intrapersonal knowledge and skills Increase interpersonal knowledge and skills Increase effective communication skills and active listening Increase aggression management techniques Increase effective problem-solving
**Phase 3: Termination phase**		
Group reflection	Discussion on whether the shared objectives were achieved	Achievement of shared objectives
Self-reflection	Was increased effectiveness achieved? Evaluation of the implementation of the model in the workshop	Achievement of own objectives Evaluation of the implementation of the model
Going forward	Implementation of the psycho-educational model in practice	Implementation of the psycho-educational model in practice in places of work

## Data collection and analysis

Phenomenological data collection methods were used during and directly after the workshop to implement the psycho-educational model. The workshop was audio-recorded and then transcribed verbatim. The participants completed reflective responses during the workshop and two reflective written feedback responses – one directly after the workshop and one 1 week after the workshop. These responses were included with the verbatim transcriptions of the recordings, as well as the presenting researcher’s (R.R.T.) field notes and observations as part of the data analysis. The single case study’s data reinforced the purpose of the model, namely, to facilitate the constructive management of experienced aggression (Rule & John 2015:5).

## Measures to ensure trustworthiness

The primary aim of trustworthiness in qualitative research is to assess the significance of the study (Lincoln & Guba 1985). Lincoln and Guba’s (1985) model for trustworthiness in line with the study’s philosophy, principles and qualitative inquiry as described in Krefting (1991:214–222), Babbie and Mouton (2011:276–278) and De Vos et al. (eds. 2011:443–444) was applied throughout the research. The criteria to ensure trustworthiness included truth value, confirmed by credibility; applicability, confirmed by transferability; consistency, confirmed by dependability; and neutrality, confirmed by confirmability. The study’s truth value – confirmed by credibility – was enhanced through triangulation (Fabio & Maree 2015:141), reflexivity (Creswell 2014:247) and structural coherence (Johnson & Rasulova 2016:15; Krefting 1991:220). Transferability was ensured through a thick description of the participants’ demographics and the description of the results. Dependability was achieved by an in-depth description of the process, structure and steps followed, supported by literature review and further enhanced by continuous peer reviewing. Confirmability was established by the chain of evidences of the research process, such as raw data, data reduction and analysis documents, process notes and reflexive notes.

### Ethical considerations

As a result of the study’s qualitative social research nature (exploratory and descriptive), strict ethical principles were adhered to. Four ethical principles were applied (South African Medical Research Council 2006:10–16), namely, autonomy, non-maleficence, beneficence and justice (Adams & Callahan 2013:n.p.; Dhai & McQuoid-Mason 2011:43–44). Ethical clearance was obtained from the Academic Ethics Committee at the university where the study was conducted (Ethical clearance #2016-086). Permission was also obtained from the management and team leader of the college of the university where the research was conducted. The participants could make a fully informed decision whether to voluntarily participate in the study (Adams & Callahan 2013:n.p.). Participants completed written and signed consent forms and were also made aware of the fact that they could withdraw from the research project at any time during the process. The researcher protected the participating university lecturers’ anonymity, confidentiality and safety by allocating patricipant numbers instead of names. Strict adherence to ethical principles was not only important for the protection of the participants but also for the feasibility of the study.

## Results

The implementation of the psycho-educational model in a workshop to facilitate constructive management of experienced aggression sought an answer to the question ‘what can be done to support university lecturers to constructively manage experienced aggression?’ The model implementation process established a single case study at a university in South Africa (Creswell 2013:97; Gustafsson 2017:11; Heale & Twycross 2018:7). Rule and John (2015:3) clarified a case study to be distinctive and specific. In this study, the single case study was an instrumental case study selected to evaluate the implementation of the developed psycho-educational model in a workshop. The single case study’s data reinforced the purpose of the model, namely, to facilitate constructive management of experienced aggression (Rule & John 2015:5).

### Findings: Workshop group size

Five university lecturers participated in the workshop. The size of the group increased the possibility for all participants to engage in and enhance the significance of the data recovered during the workshop (Cohen, Manion & Morrison 2011:156–157; Leedy & Ormrod 2010:146–147). The size of the group made it possible for participants to share cognitive patterns of thought and/or behaviour to construct and reconstruct ideas through effective communication (Matteson 2010:37–38). This then culminated in better shared understanding, newly shaped patterns and shared cognition in the group. Additionally, the size of this group allowed for greater active involvement and unique effective conversations and learning (Brame & Biel 2015:n.p.; Seeds for Change 2010:9).

### Findings: Phase 1 – Relationship phase

The relationship phase of the model served to create a safe space for interaction and conversation. It established the starting point for a trusting relationship, active participation and dynamic and collaborative interaction that the rest of the facilitation process depended on.

Participants found that the activities in this phase led to the building of a trusting relationship amongst the participants and between the participants and the facilitator, as evident in the following quote:

‘… We were immediately informed that everything discussed in the workshop would be kept confidential, which created space for openness and transparency.’ (Participant 1)

‘The workshop was facilitated well, nice ice-breaker and building of rapport with each other.’ (Participant 4)

According to McCallum (2010:9), trust ensures that a team can function effectively, and it also increases team members’ confidence to interact and communicate their personal opinions. The relationship phase of the model also assisted university lecturers to progress from unawareness of and not understanding the problem of experienced aggression in higher education to awareness and understanding of the phenomenon. They also gained greater insight into the concept of destructive management of experienced aggression. The relationship phase shaped the direction of the facilitation process. The introduction of a variety of activities underpins Jacobs’ (2013:211) statement that various strategies are required in facilitation because of the multidimensionality of the ‘facilitation’ process. Effective communication and understanding of what aggression and destructive management are, as well as the collaborative interaction, discovery and conversation, assisted the participants to move to the second phase of the model, namely, the working phase. Participants remarked:

‘I realised how disruptive unattended aggression experienced by individuals can be in the workplace. It is untold recipe for discouragement, disappointment, disruption of productivity and relational distances between colleagues.’ (Participant 5)

‘Acknowledging that we don’t know how to manage aggression.’ (Participant 1)

### Findings: Phase 2 – Working phase

Considering the outcome of the implementation of the psycho-educational model to increase university lecturers’ effectiveness in managing experienced aggression constructively, the main objective of the working phase was to ‘facilitate constructive management’ of experienced aggression. The tasks and activities contributed to achieve this aim. The concept ‘constructive’ is defined in this research as being helpful to the university lecturers and promoting the lecturers’ development. In the participants’ responses, the understanding of ‘constructive’ was reflected in remarks such as to promote, improve and enhance, thus confirming that the essential attributes are helpful and promote development:

‘Promote healthy relationships with colleagues and peers and students.’ (Participant 3)

‘Improve interpersonal communication amongst members will enhance productivity and morale.’ (Participant 5)

The concept ‘management’ was defined as to deal with and control. During the participants’ understanding and meaning-making of the concept ‘management’, these two essential attributes were confirmed by their responses:

‘We said to deal with to put it into perspective …’ (Participant 2)

‘I think you control aggression … Control it by being fair …’ (Participant 3)

After the discovery and self-directed learning of the concepts ‘constructive management’, the tasks and activities implemented in the workshop focussed on knowledge and soft skills development on four levels: intrapersonal skills, interpersonal skills, communication skills and conflict management skills. Participants found the discovery of new knowledge and soft skills insightful, developmental and effective for application in their places of work to constructively manage experiences of aggression. Participants’ increased effectiveness was expressed in statements of their willingness to acknowledge and be open to other perceptions, reflect and respond to aggressive behaviour rather than react to it, improve their communication with and listening to other people and being assertive and taking control of their own emotions and experiences of aggression. The participants said:

‘Be fair when you’re in that situation and manage your own behaviour.’ (Participant 4)

‘… Should understand other co-workers’ feelings and emotions as this will improve how to interact and communicate in the workplace.’ (Participant 5)

In conclusion, participants stated that it might be beneficial to implement the model in a 2-day to two-and-a-half-day workshop. The longer workshop time would allow in-depth discovery of and discussion on the types of aggression in higher education and deeper learning of the soft skills knowledge development and comprehensive accomplishment thereof necessary for the constructive management of experienced aggression. Participants’ recurring feedback on the need for greater development, knowledge and skills to manage experienced aggression constructively demonstrates the importance of softer skills training for university lecturers to increase their effectiveness on a personal and professional level:

‘Need more time to discuss examples and real-life experiences. Find out what we as lecturers have in common.’ (Participant 2)

### Findings: Phase 3 – Termination phase

The termination phase allowed reflection on the process, essential attributes, skills and the value of the model for participants. Self-reflection on the significance of the workshop experience answered the open-ended question, ‘how was the workshop for you?’ Participants believed their expectations of the workshop and the model were met after they understood the concept ‘aggression’ and constructive management of experienced aggression in higher education. The participants’ responses underpinned that the model and workshop knowledge and skills discovery and self-directed learning increased their effectiveness in managing experiences of aggression more constructively. Moreover, the participants believed that they gained increased effectiveness in applying the acquired knowledge and skills in their workplaces to manage experiences of aggression more constructively. The participants stated that the self-reflection time in the termination phase was important for them to end the facilitation process. Examples of these responses are the following:

‘The workshop was very informative, and I have learnt a lot about the concepts of aggression and how to manage it.’ (Participant 2)

‘The intervention and efforts made to move away from the aggressive behaviour and new interpersonal skills has been acquired.’ (Participant 4)

## Limitations of the study

It is important to recognise that there are limitations of the study. The study only included one specific faculty of a university and not all faculties. The psycho-educational model’s implementation in a workshop was also influenced by challenges in higher education. University lecturers have vast academic responsibilities, which include teaching, research, students’ winter schools and conference attendance. These responsibilities, as well as the university academic holidays, made it difficult to finalise a specific date and time in their calendar that suited all voluntary participants. Thus, the workshop had to fit into a half-day. It would have been better to implement the model in a day-and-a-half workshop to be able to go into deeper discovery and discussions of the four competencies. Even with the workshop only being a half-day, one of the participants had to leave and return during the workshop to attend another important departmental meeting. The presenting researcher, as the facilitator, accommodated this gap by meeting with the specific participating university lecturer and concluding the workshop again from the working phase to the termination phase on a one-on-one basis.

## Recommendations

It is recommended that university lecturers’ experiences of aggression should be acknowledged as a reality to be constructively managed. Therefore, training and development of knowledge and skills to ‘manage’ experiences of aggression ‘constructively’ is essential. Workshops to implement the psycho-educational model could be integrated into all orientation programmes for newly appointed staff members as a preventative measure of workplace aggression. The psycho-educational model could also be implemented as part of the university staff’s ongoing development, talent management focus and policies.

## Conclusion

This study has theoretical and practical value in the field of psycho-education and for universities and higher education overall. The contribution of this article is the implementation of a psycho-educational model for university lecturers to facilitate the constructive management of experienced aggression. The implementation and evaluation of the model, as a conceptual framework of reference for university lecturers, set the stage for deliberate examination, description and critical reflection of the model in a workshop. Based on the data collection and analysis of this implementation process, the facilitator is convinced that the workshop and the workshop process created a safe and successful facilitative space that increased the participants’ effectiveness in managing experiences of aggression more constructively. Additionally, critical reflection on the implementation of the model, without biased views of meaning (Chinn & Kramer 2015:220–222), underpinned that the model, the identification of knowledge and skills, and self-directed learning processes are helpful in promoting university lecturers’ development and increasing their effectiveness in dealing with and constructively managing experienced aggression.
